# P21 facilitates macrophage chemotaxis by promoting CCL7 in the lung epithelial cell lines treated with radiation and bleomycin

**DOI:** 10.1186/s12967-023-04177-5

**Published:** 2023-05-09

**Authors:** Xinglong Liu, Liang Zeng, Yuchuan Zhou, Xinrui Zhao, Lin Zhu, Jianghong Zhang, Yan Pan, Chunlin Shao, Jiamei Fu

**Affiliations:** 1 Shanghai Institute of Infectious Disease and Biosecurity, and Institute of Radiation Medicine, Shanghai Medical College, Fudan University, Shanghai, 200032 China; 2 Institute of Radiation Medicine, Shanghai Medical College, Fudan University, Shanghai, 200032 China; 3 Department of Radiation Oncology, Shanghai Pulmonary Hospital, School of Medicine, Tongji University, Shanghai, 200433 China

**Keywords:** Radiation-induced lung injury, Bleomycin-induced pulmonary fibrosis, p21, CCL7, Macrophage chemotaxis

## Abstract

**Background:**

Interstitial lung diseases (ILDs) can be induced and even exacerbated by radiotherapy in thoracic cancer patients. The roles of immune responses underlying the development of these severe lung injuries are still obscure and need to be investigated.

**Methods:**

A severe lung damage murine model was established by delivering 16 Gy X-rays to the chest of mice that had been pre-treated with bleomycin (BLM) and thus hold ILDs. Bioinformatic analyses were performed on the GEO datasets of radiation-induced lung injury (RILI) and BLM-induced pulmonary fibrosis (BIPF), and RNA-sequencing data of the severely damaged lung tissues. The screened differentially expressed genes (DEGs) were verified in lung epithelial cell lines by qRT-PCR assay. The injured lung tissue pathology was analyzed with H&E and Masson’s staining, and immunohistochemistry staining. The macrophage chemotaxis and activity promoted by the stressed epithelial cells were determined by using a cell co-culture system. The expressions of *p21* in MLE-12 and Beas-2B cells were detected by qRT-PCR, western blot, and immunofluorescence. The concentration of CCL7 in cell supernatant was measured by ELISA assay. In some experiments, Beas-2B cells were transfected with *p21*-siRNA or *CCL7*-siRNA before irradiation and/or BLM treatment.

**Results:**

After the treatment of irradiation and/or BLM, the inflammatory and immune responses, chemokine-mediated signaling pathways were steadily activated in the severely injured lung, and *p21* was screened out by the bioinformatic analysis and further verified to be upregulated in both mouse and human lung epithelial cell lines. The expression of P21 was positively correlated with macrophage infiltration in the injured lung tissues. Co-culturing with stressed Beas-2B cells or its conditioned medium containing CCL7 protein, U937 macrophages were actively polarized to M1-phase and their migration ability was obviously increased along with the damage degree of Beas-2B cells. Furthermore, knockdown *p21* reduced CCL7 expression in Beas-2B cells and then decreased the chemotaxis of co-cultured macrophages.

**Conclusions:**

P21 promoted CCL7 release from the severely injured lung epithelial cell lines and contributed to the macrophage chemotaxis in vitro, which provides new insights for better understanding the inflammatory responses in lung injury.

## Introduction

Interstitial lung diseases (ILDs), as a group of heterogeneous abnormalities, are primarily characterized by inflammation or fibrosis within the pulmonary interstitium. The classical physiopathologic change features as impaired gas exchange that is clinically manifested by dyspnea, restrictive ventilation impairment and hypoxemia. In terms of etiology, ILDs can be induced by a large number of serious stresses, such as toxins, radiation, occupation-related dust, inhaled particles and a series of autoimmune diseases. Elderly patients with ILDs are usually at high risk of lung cancer. Meanwhile, interstitial pneumonitis and pulmonary fibrosis are common lung toxicity associated with cancer treatment strategies, including chemotherapy, targeted therapy, immunotherapy and radiotherapy. Especially, when multiple treatments are performed, the preexisting lung diseases may be exacerbated and lead to breathlessness, respiratory failure and even death [1]. However, the molecular mechanisms of the severe lung injury caused by various stimuli have not been clearly revealed, and there is still a lack of effective early diagnostic biomarkers and molecular targeted therapy.

In order to better understand the development mechanism of lung pathology, numerous animal models of lung injury have been established [2, 3]. The disease models of bleomycin (BLM)-induced pulmonary fibrosis (BIPF) and radiation-induced lung injury (RILI) are commonly used in oncology since they can better mimic the successive progression of pneumonitis and lung fibrosis after drug treatment or radiotherapy [4]. Moreover, the potential commonality and differential susceptibility in the pathogenesis have received more attention nowadays, including the critical role of alveolar epithelial cells (AECs) in the development of lung injury. Single-cell transcriptome analysis demonstrated that AECII cells were essential in the regeneration of alveolar structure [5]. Dysfunctional AECs that experienced apoptosis or senescence contributed to the recruitment and infiltration of immune cells. Besides, based on microenvironment changes, macrophages are highly plastic and dynamic and play important roles in the pro-inflammatory or anti-inflammatory responses to lung injury [6]. Although the intercellular communication between AECs and macrophages has been well recognized in maintaining lung homeostasis, the relative crucial signaling networks in specific diseases are still poorly understood [7].

Cdkn1a/P21 is an important endogenous cyclin-dependent kinase (CDK) inhibitor, whose functions are widely related to DNA damage and repair, cell cycle arrest, transcriptional regulation, cellular differentiation, senescence and apoptosis. Interestingly, it has recently been found that *p21* has multiple aspects in the regulation of immune system according to its cellular context and cytoplasmic localization [8]. The status of *p21* in macrophages was considered as a suppressor of inflammation. Autoimmunity diseases relying on macrophage activation, such as arthritis, could be established in *p21*-deficient mice. On the contrary, some studies reported that *p21* could promote inflammation of pneumonia, septic shock, pulmonary fibrosis, atherosclerosis and autoimmune pancreatitis by regulating the production of ACEII, immune cells and cytokines [9, 10]. CCL7, also known as monocyte chemoattractant protein-3 (MCP-3), is a member of chemokine subfamily that features as a chemoattractant for various leukocytes. Abnormal CCL7 level in tumor microenvironment is associated with tumorigenesis and promotes tumor invasion and metastasis [11]. However, little is known about the exact role of CCL7 in lung toxicity.

Our previous studies have found that the intercellular crosstalk between irradiated lung epithelial cells and bystander macrophages contributes to the aggravated injury of epithelial cells [12, 13]. This process works by enhancing the production of cytokines interleukin (IL)-6 and tumor necrosis factor (TNF)-α regulated by the signaling pathways of mitogen-activated protein kinases (MAPKs) and nuclear factor (NF)-κB. However, it remains ill-defined that how the injured epithelial cells could be recognized and monitored by the immune system. By using different lung injury animal models of RILI, BIPF and the severe lung damage model established by one-shot irradiation (IR) with pre-existed ILDs, and a cell co-culture model of lung epithelial cells and macrophages, this study investigated the molecular mechanism underlying the development of lung pathological injury, including the roles of *p21* and chemokine CCL7 in the chemotaxis and infiltration of macrophages.

## Materials and methods

### Functional enrichment analysis of microarray data

Series matrix files of two microarray datasets (GSE41789 and GSE37635) were obtained from the Gene Expression Omnibus (GEO) database [14]. Dataset GSE41789 was collected from the platform GPL1261 (Affymetrix Mouse Genome 430 2.0 Array) that included 45 mouse lung tissue samples (10-week-old female C57Bl/Ncr mice) with/without radiation in an age-matched cohort. Dataset GSE37635 was collected from that platform GPL6885 (Illumina MouseRef-8 v2.0 expression beadchip) that included 38 mouse lung tissue samples (10–12-week-old female C57BL/6 J mice) with/without BLM-instillation.

To recognize the differentially expressed genes (DEGs) in the lung tissues of mice, the lung samples at 8 weeks after 17.5 Gy IR (GSE41789: GSM1024338-GSM1024340, GSM1024344-GSM1024346) and the lung samples at 4 weeks after BLM instillation (GSE37635: GSM9863383-GSM986390, GSM986391-GSM986398) were selected to carry out gene expression analysis. The Bioconductor package limma (v3.46.0) was applied in software R (v4.0.3) to conduct statistical analysis of two defined group [15]. DEGs were identified using the adjusted p < 0.05 and |log_2_ (fold change)|> 0.585 as the screening thresholds.

The functional enrichment analysis conducted by using Gene Ontology (GO) and Kyoto Encyclopedia of Genes and Genomes (KEGG) enables us to understand the cellular distribution and the main functions of DEGs, which was performed in the Database for Annotation, Visualization, and Integrated Discovery (DAVID) (v6.8) [16], and p < 0.05 was considered to be of statistical significance. The visualization of analysis result was run with R package ggplot2 (v3.3.5) [17].

### Establishment of severe lung injury mouse model of ILDs

Six-to-eight-week-old C57BL/6 J male mice (GemPharmatech, Jiangsu, China) were anaesthetized for single intratracheal administration of BLM sulfate (Meilunbio, Liaoning, China; 2 mg/kg body weight) in 40 μL of 0.9% saline [18]. Mice in the control group were administrated with the same volume of saline. Four weeks after BLM treatment, the right thorax of mice under anesthesia was exposed to a single dose of 16 Gy or 0 Gy X-rays (2.0 Gy/min) (X-RAD 320, Precision X-Ray Inc., North Branford, CT, USA), which was adjusted from our previous study [19]. Mice were grouped according to the treatment: saline + sham-IR (Control), saline + IR (RILI), BLM-instillation + sham-IR (BIPF), and BLM-instillation + IR (BIPF + RILI). A portion of lung tissues at 1 and 4 weeks after IR with or without BLM treatment were cryopreserved at – 80 ℃ for RNA sequencing assay, and the other tissues were fixed in 4% paraformaldehyde for H&E staining and Masson’s staining. All animal experimental protocols were authorized by the Animal Ethics Committee of Fudan University.

### RNA-Sequencing data collection and analysis

RNA extracted from each mouse lung sample was sequenced on the Illumina Novaseq platform with sequencing libraries generated, index codes added, and clusters generated. Paired-end reads of 150 bp were generated, and the reads containing adapters, poly-N and low-quality ones were weeded out using TrimGalore software (v0.6.6) [20]. Clean data were mapped to the mouse reference genome (mm10) with TopHat software (v2.1.1) [21] and then evaluated using Cufflinks software (v2.2.1) [22]. Quantitative differentiation of gene expressions between the two groups were analyzed using the Cuffdiff program of Cufflinks. DEGs of RNA-sequencing (RNA-seq) data were filtered with threshold adjusted p < 0.05 and |log_2_(fold change)|> 0.585. Gene Set Enrichment Analysis (GSEA) was performed using the clusterProfier package (v3.18.1) [23].

### H&E staining and Masson’s staining

Lung tissues isolated from mice in different groups were fixed in 4% paraformaldehyde for 24 h, dehydrated with graded ethanol, embedded with paraffin according to the standard procedures, and then cut into 4 μm thickness. H&E staining was conducted with hematoxylin and eosin (G1005, Servicebio, Hubei, China) for 5 min after dewaxing and hydration. Masson’s staining was performed using a staining Kit (G1006, Servicebio). Images in different groups were captured using a microscope (Olympus IX51, Tokyo, Japan).

### Cell culture and treatments

Mouse lung epithelial cell line MLE-12, human lung epithelial cell line Beas-2B, and human macrophage cell line U937 were obtained from Shanghai Cell Bank of the Chinese Academy of Sciences (Shanghai, China). MLE-12 cells and Beas-2B cells were cultured in Dulbecco’s modified Eagle’s medium with high glucose (DMEM) (Gibco, Thermo Fisher Scientific, Waltham, MA, USA). U937 cells were cultured in RPMI-1640 medium (Gibco). All medium was supplemented with 10% fetal bovine serum (FBS) (Gibco), 100 mg/mL streptomycin, and 100 units/mL penicillin (Gibco). Cells were incubated in a water-jacketed incubator (Thermo Fisher Scientific) at 37 ℃ in 5% CO_2_. To obtain adherent M0 macrophages, U937 cells were treated with 100 ng/ml phorbol 12-myristate 13-acetate (PMA) (HY-18739, MCE, Shanghai, China) for 24 h.

For BLM treatment, cells at 70% confluence were washed twice with sterilized phosphate buffered saline (PBS) and cultured in the corresponding medium containing 10 μg/mL BLM for 6 h. For IR treatment, cells were exposed to 6 Gy X-rays (0.833 Gy/min) at room temperature. MLE-12 and Beas-2B cells were divided into four groups: sham-IR + PBS (Ctrl), IR, sham-IR + BLM (BLM), and BLM + IR (B + IR).

### Quantitative real-time PCR assay

Quantitative real-time PCR (qRT-PCR) was used to verify the RNA expression levels of DEGs. An RNA Easy Fast Tissue/Cell Kit (Tiangen, Beijing, China) was used to extract total cellular RNA according to the manufacturer’s protocol. Then 2 μg of RNA was reversely transcribed into cDNA using a FastKing RT Kit (Tiangen). qRT-PCR was performed in 20 μL reaction reagent using SuperReal PreMix Plus (SYBR Green) (Tiangen) on the MX3000P platform (Agilent, Santa Clara, CA, USA). The relative quantification of gene expression was assessed by comparative threshold cycle (Ct), and the fold change of target genes was calculated by the 2^−ΔΔCt^ method. The specific primers of the target genes (mouse: *Lcn2*, *Cdkn1a/p21*, and *Lrg1*; human: *LCN2*, *CDKN1A/p21*, *LRG1*, *CCL2*, *CCL7*, *CXCL5*, *CXCL9*, *CXCL10*, *CXCL14*, and *CXCL16*) and the reference gene (*β-actin*) are listed in Table 1.

**Table 1:** Primers for genes in qRT-PCR assay

Species	Gene	Primers (5′-3′)	
Mus musculus	*Lcn2*	Forward	GGGAAATATGCACAGGTATCCTC
		Reverse	CATGGCGAACTGGTTGTAGTC
	*Cdkn1a/p21*	Forward	CCTGGTGATGTCCGACCTG
		Reverse	CCATGAGCGCATCGCAATC
	*Lrg1*	Forward	TACAGCACCTGGATATGTTGGA
		Reverse	GTTGTGGGAGATGTCGAAGCC
	*β-actin*	Forward	AGAAGCTGTGCTATGTTGCTCTA
		Reverse	AGACAGCACTGTGTTGGCATA
Homo sapiens	*LCN2*	Forward	TACCTCGTCCGAGTGGTGAG
		Reverse	GGGACAGGGAAGACGATGTG
	*CDKN1A/p21*	Forward	TTGTCACCGAGACACCACTG
		Reverse	AGTGGTAGAAATCTGTCATGCT
	*LRG1*	Forward	TGTCAACCACCTGCCGAAAT
		Reverse	GAGAGGCTTTCCAGCCCATT
	*β-ACTIN*	Forward	AGGATTCCTATGTGGGCGAC
		Reverse	GATAGCACAGCCTGGATAGCAA
	*CCL2*	Forward	CAGCCAGATGCAATCAATGCC
		Reverse	TGGAATCCTGAACCCACTTCT
	*CCL7*	Forward	ATCCCTAAGCAGAGGCTGGA
		Reverse	GTCCTGGACCCACTTCTGTG
	*CXCL5*	Forward	AGCTGCGTTGCGTTTGTTTAC
		Reverse	TGGCGAACACTTGCAGATTAC
	*CXCL9*	Forward	CCAGTAGTGAGAAAGGGTCGC
		Reverse	AGGGCTTGGGGCAAATTGTT
	*CXCL10*	Forward	GTGGCATTCAAGGAGTACCTC
		Reverse	TGATGGCCTTCGATTCTGGATT
	*CXCL14*	Forward	CGCTACAGCGACGTGAAGAA
		Reverse	GTTCCAGGCGTTGTACCAC
	*CXCL16*	Forward	GACATGCTTACTCGGGGATTG
		Reverse	GGACAGTGATCCTACTGGGAG

### Western blot assay

Protein expression was measured by western blot assay. Total cellular protein extraction was performed using RIPA buffer (Beyotime, Shanghai, China) with loading buffer containing protease inhibitors. An equal amount of protein was then separated by 12.5% sodium dodecyl sulfate–polyacrylamide gel electrophoresis (SDS-PAGE) at a constant voltage, and transferred to a polyvinylidene difluoride (PVDF) membrane (Millipore, Burlington, MA, USA) using a wet method for further antibody binding reactions. After blocking with 50 mL 5% non-fat milk in Tris-buffered saline/Tween 0.05% (TBST) for 1.5 h, the membrane was incubated with primary antibodies of anti-P21 (1:1000, A19094, ABclonal, Hubei, China) and anti-α-tubulin (1:1000, AF0001, Beyotime) at 4 ℃ overnight. After incubating with proper second antibodies (1:3000, Beyotime) for 1.5 h, the proteins were finally detected using an ECL kit (Millipore) and analyzed using the ChemiDoc XRS system (Bio-Rad Laboratories, Hercules, CA, USA).

### CCK-8 assay

The CCK-8 assay was performed to detect cell viability using the Enhanced Cell Counting Kit-8 (CCK-8) (C0042, Beyotime) following to the manufacturer’s instructions. Briefly, Beas-2B cells were seeded in the 96-well plate (1 × 10^4^ cells in 100 μL/well and then treated with IR and/or BLM when the cells approached to 70% confluence. After 6 h, the medium was renewed and the cells were cultured for another 24 h. Next, 10 μL CCK-8 solution was added into each well and the plate was incubated for 2 h. The OD value was detected at a wavelength of 450 nm.

### Immunofluorescence staining and imaging of cells

After fixation with 4% paraformaldehyde for 10 min, cells were permeabilized with 0.5%Triton X-100 (P0096, Beyotime) for 10 min and blocked with QuickBlock™ Blocking Buffer for Immunol Staining (P0260, Beyotime) for 1 h. Cells were then incubated with P21 primary antibody (1:500, A19094, Abclonal) at 4 ℃ overnight and Alexa Fluor 555-conjugated secondary antibody (1:1500, A0453, Cell Signaling Technology, Danvers, MA, USA) for 1.5 h at room temperature. Cell nuclei were stained with DAPI (C1006, Beyotime) for 10 min. Immunofluorescence images were captured using an ImageXpress Micro 4 screening system (Molecular Devices, Sunnyvale, CA, USA) and the percentage of positive cells was analyzed with the ImageJ software (v1.8.0, National Institutes of Health, USA).

### Immunohistochemistry staining for lung tissue

Mouse lung tissue was fixed in 4% paraformaldehyde for 24 h, followed by gradient dehydration, paraffin embedding, sliced, and antigen retrieval. The tissue sections were then blocked with BSA (G5001, Servicebio) and incubated with primary antibodies anti-P21 (1:400, GB11153, Servicebio), anti-pro-SPC (1:2000, ab211326, Abcam, Cambridge, UK), and anti-F4/80 (1:5000, Abcam), and counterstained with hematoxylin. The images were captured using a Leica DFC7000T microscope and the positive area was analyzed using ImageJ software.

### Cell co-culture

Cell co-culture system was conducted in a six-well plate with insert chamber (0.4 μm pores, Corning Inc., Corning, NY, USA) according to the protocol adapted from our previous study [12]. PMA-pretreated U937 cells (1 × 10^6^ cells/well) were seeded into the upper insert chamber, and Beas-2B (5 × 10^5^ cells/well) treated with 6 Gy X-rays and/or 10 μg/mL BLM for 6 h were well washed and then seeded in the lower chamber. After 24 h of co-culture, U937 cells were digested and collected for flow cytometry assay described below.

### Flow cytometry assay

After being co-cultured with Beas-2B cells for 24 h, U937 cells were digested, washed, and resuspended in 100 μL staining buffer (554554, BD pharmingen, Franklin Lakes, NJ, USA). The non-specific FC receptors were blocked with Human BD FC Block (0.5 mg/mL, 56429, BD pharmingen) to avoid false positive. The cells were then incubated with antibodies PE-CY7-anti-CD68 (561128, BD pharmingen) and Alexa Flour 647-anti-CD163 (562669, BD pharmingen) at 4 ℃ in the dark condition. Next, the cells were incubated with antibody PerCP/Cyanine 5.5-anti-CD86 (333814, Biolegend, San Diego, CA, USA) under the same conditions after fixation and permeation. The fluorescence intensity of 10,000 cells in different groups was detected by a flow cytometry (Beckman CytoFLEX, Pasadena, CA, USA).

### Cell migration assay

Cell migration assay was performed in a 24-well plate with insert chamber (8 μm pores, Corning). PMA-pretreated U937 cells (6 × 10^5^ cells/well) with DiO staining (C1993S, Beyotime) were suspended in 200 μL serum-free RPMI-1640 medium and seeded into the upper insert chamber. After 24 h of co-culture with Beas-2B (5 × 10^5^ cells/well) treated with 6 Gy X-rays and/or 10 μg/mL BLM for 6 h in the lower chamber, the migrated U937 cells on the reverse side of insert chamber were photographed under a fluorescence microscopy.

Additionally, to identify whether macrophage chemotaxis could be induced by inflammatory factors (such as CCL7) secreted by stressed Beas-2B cells, the conditional medium (CM) was collected from Beas-2B cells (5 × 10^5^ cells/well) incubated for 24 h after IR and/or 6 h BLM-treatment with/without *p21* or *CCL7* RNA interference. 600 μL CM or the complete medium containing active CCL7 protein (0, 50 and 100 pg/mL) was added into the lower chamber of a 24-well plate where an insert dish containing U937 macrophages was conjugated. After 48 h, U937 cells migrated through the membrane to the reverse side of the insert dish were fixed with 4% paraformaldehyde for 10 min and then stained with 1% crystal violet for 15 min, then five randomly selected fields for each chamber were observed under a microscope to count the number of migrated cells.

### ELISA assay

Beas-2B cells treated with IR and/or BLM were continually incubated for 24 h, then cell supernatants were centrifuged at 3000 rpm for 10 min. The chemokine CCL7 in the supernatants was then measured using the Human CCL7/MCP-3 ELISA Kit (U96-1543E, YOBIBIO, Shanghai, China) according to the manufacturer's instructions. The OD value was detected at a wavelength of 450 nm and the concentration of CCL7 protein was calculated according to the standard curve.

### RNA interference

Three *p21*-targeting small interfering RNAs (siRNAs) (si-*p21*-1, si-*p21*-2, si-*p21*-3) and three *CCL7*-targeting siRNAs (si-*CCL7*-1, si-*CCL7*-2, si-*CCL7*-3) for human cells and their negative control (si-NC) were supplied by Ribobio (Guangzhou, China). Beas-2B cells were transfected with 50 nM siRNAs or si-NC using riboFECTTM CP Reagent (Ribobio) for 24 h. According to the optimal interference efficiency, si-*p21*-2 and si-*CCL7*-3 were used for subsequent experiments. The sequences of the siRNAs were as follows: si-*p21*-1 (sense: 5′-GAT GGA ACT TCG ACT TTGT-3′), si-*p21*-2 (sense: 5′-AGA CCA TGT GGA CCT GTCA-3′), si-*p21*-3 (sense: 5′-GAG ACT CTC AGG GTC GAAA-3′); si-*CCL7*-1 (sense: 5′-GAA GCA CCT GGA CAA GAAA-3′), si-*CCL7*-2 (sense: 5′-ACC TGC TGC TAC AGA TTTA-3′), si-*CCL7*-3 (sense: 5′-GAA GTG GG TCC AGG ACTTT-3′).

### Correlation analysis of CCL7 and immune infiltration level

Correlation of *CCL7* gene expression with the immune infiltration level was analyzed using the Immune Association-Gene module of TIMER2.0, a comprehensive resource for systematic analysis of immune infiltrates across multiple cancer types [24].

### Statistical analysis

The experimental data from at least three replicates were presented as mean ± standard deviation (SD) for normal distribution data and median with interquartile range (IQR) for non-normal data. We assessed normality of data using the Shapiro–Wilk normality test with software GraphPad Prism 7 (San Diego, CA, USA). If data was normally distributed, comparative statistical analysis was performed by Student’s t-test between two groups and by one-way analysis of variance (ANOVA) between more than two groups. If data was not normally distributed, non-parametric test was performed. *P* < 0.05 was considered statistically significant unless otherwise indicated.

## Results

### Functional enrichment of DEGs in the mouse models of RILI and BIPF

**Fig. 1:**
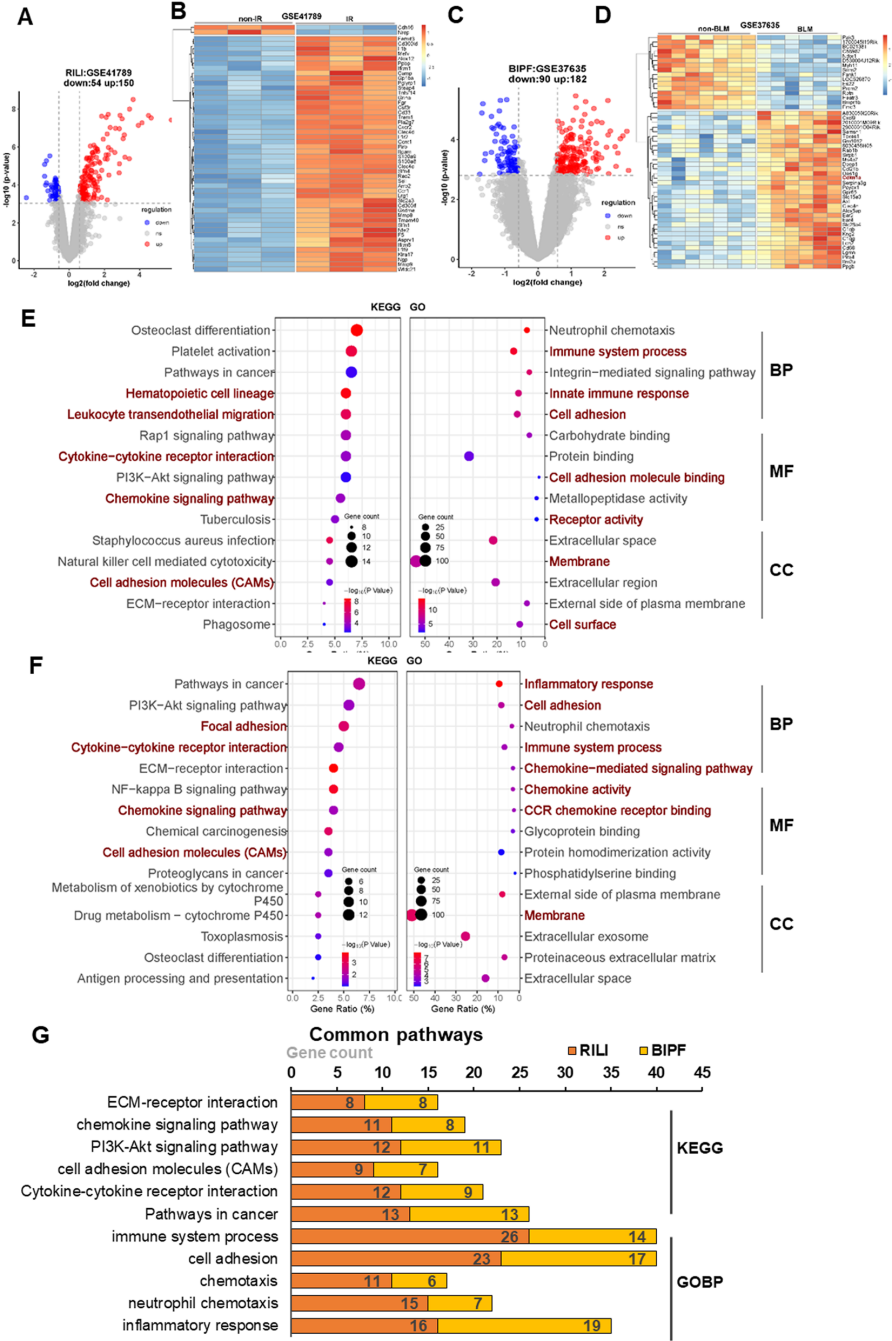
Functional enrichment analysis of DEGs in RILI and BIPF model mice. **A** Volcano plot of the genes in dataset GSE41789. Red dots represent the upregulated genes (up), blue dots represent the downregulated genes (down), and gray dots represent the genes with no significant (ns) changes in the irradiated samples. Adjusted p-value < 0.05 with the threshold |log_2_(Fold Change)|> 0.585 is considered significant. **B** Heatmap of top fifty DEGs between irradiated group and its nonirradiated control in the dataset GSE41789 with expression value normalized from – 1–1.** C** Volcano plot of the genes in the dataset GSE37635. Red dots represent the upregulated genes (up), blue dots represent the downregulated genes (down), and gray dots represent no the genes with no significant (ns) changes in BLM-instillation samples. Adjusted p-value < 0.05 with the threshold |log_2_(Fold Change)|> 0.585 is considered significant. **D** Heatmap of the top fifty DEGs between BLM-treated group and its control in the dataset GSE41789 with expression value normalized from – 1–1.** E** Bubble plot showing the top fifteen KEGG pathways and top five terms in BP, MF and CC, respectively, in GO analysis enriched by the DEGs in the dataset GSE41789.** F** Bubble plot showing the top fifteen KEGG pathways and top five terms in BP, MF and CC, respectively, in GO analysis enriched by the DEGs in the dataset GSE37635.** G** Bar plot showing the common KEGG pathways and GOBP pathways enriched by the DEGs in datasets GSE41789 and GSE37635

To identify the hub genes in the injured lung of mice exposed to IR or BLM, we analyzed the microarray data in the dataset GSE41789 and dataset GSE37635, where total lung tissue RNA was extracted at 8 weeks after 17.5 Gy IR or 4 weeks after BLM treatment. The volcano plot illustrated that there were 150 upregulated genes and 54 downregulated genes in the irradiated samples in comparison with non-irradiated ones (Fig. 1A). The cluster analysis result of top fifty DEGs between two groups was shown in a heatmap (Fig. 1B).

For the BLM-induced lung damage, six biological replicates of BLM-instillation lung tissues and seven replicates of sanitary saline-instillation tissues were extracted and analyzed. The volcano plot displayed 182 upregulated genes and 90 downregulated genes in the BLM treatment group in comparison with the control group (Fig. 1C), and the heatmap of the cluster analysis of top fifty DEGs between these two groups was shown in Fig. 1D.

We then performed functional analysis of the DEGs. For the IR group, Fig. 1E illustrates top fifteen pathways in the KEGG analysis and top five terms in the GO analysis including biological process (BP), molecular function (MF) and cellular component (CC). Clearly, some important pathways including chemokine signaling pathway, cytokine-cytokine receptor interaction, and cell adhesion molecules (CAMs) were involved in RILI. Correspondingly, these pathways were clustered in the immune system process and cell adhesion of BP, and the cell adhesion molecule binding and receptor activity of MF. Therefore, the chemotaxis of different immune cells and the responses of immune system were crucial in the lung damage induced by IR.

In line with the animal model of RILI, KEGG pathway analysis demonstrated that the chemokine signaling pathway, cytokine-cytokine receptor interaction and CAMs were also involved in the BIPF (Fig. 1F). Correspondingly, the inflammatory responses, immune system process and chemokine-mediated signaling pathway were steadily enriched in the GO analysis.

Furthermore, we analyzed the common pathways between RILI and BIPF models, and found that they had considerable commonalities in the pathophysiological processes. Inflammation of immune system, cell adhesion and chemotaxis-related signaling pathways were critically involved in the molecular mechanisms of lung injury after either IR or BLM treatment (Fig. 1G).

### Signaling pathways in the mouse model with severe lung injury

**Fig. 2:**
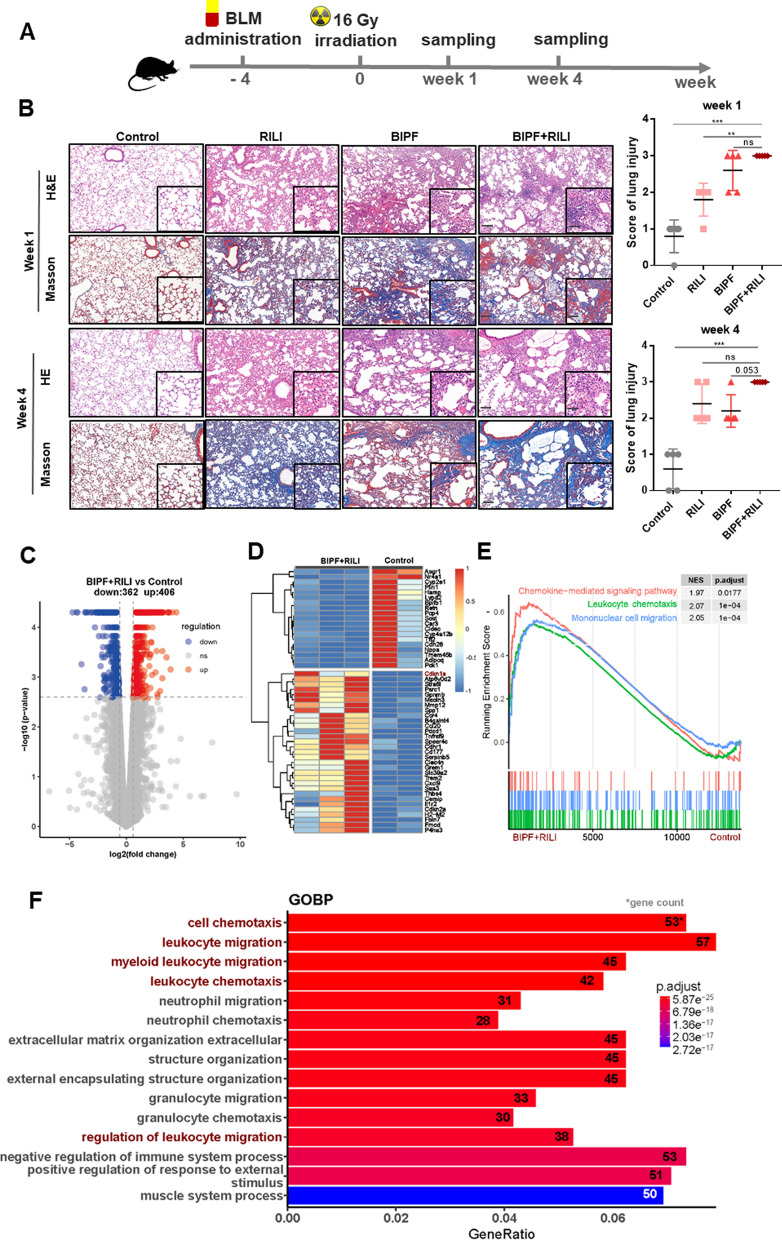
Functional enrichment analysis of DEGs in the BIPF + RILI model mice. **A** The schedule for the establishment of BIPF + RILI mouse model. **B** H&E and Masson’s staining images (left) and the score of lung injury (right) at week 1 and week 4 after irradiation. n = 5, scale bar = 50 μm, magnifying scale bar = 25 μm; **p < 0.01, ***p < 0.0001, ns: no significant. **C** Volcano plot of the genes in RNA-seq data from BIPF + RILI model at week 1 after irradiation. Red dots represent the upregulated genes (up), blue dots represent the downregulated genes (down), and gray dots represent the genes no changes (ns) in both BLM-instillation and irradiated samples. Adjusted p-value < 0.05 with the threshold |log_2_(Fold Change)|> 0.585 is considered significant. **D** Heatmap of the top fifty DEGs between BIPF + RILI model and its control with expression value normalized from – 1–1. **E** GSEA enrichment pathways of RNA-seq data. **F** Bar plot showing the top fifteen GOBP pathways enriched by DEGs of RNA-seq data

To further investigate the critical genes engaged in the lung injury, a severe lung damage mouse model (BIPF + RILI) was established. Briefly, when the phenotype of pulmonary fibrosis disease was stable at 4 weeks after BLM instillation, the right thorax of mice was irradiated with 16 Gy X-rays (Fig. 2A). At 1 and 4 weeks after IR, the lung tissues from each group of control, RILI, BIPF, and BIPF + RILI were collected for further investigation. H&E and Masson’s staining of tissue pathological structure showed that the infiltration of inflammatory cells and thickened alveolar septum in the lung tissues were obviously increased at week-1 after IR, and the myofibroblast hyperplasia and collagen deposition had a high level at week-4 after IR (Fig. 2B). For the BLM-treated mice, the typical fibrosis phenotype with interstitial infiltration of inflammatory cells and deposition of collagen fibers were observed at week-5 after BLM treatment (i.e. week-1 after sham-IR), and stable lung injury was mainly featured with destroyed alveoli and honeycomb cysts at week-8 after BLM administration. For the combination treatment of BLM and IR, more serious lung injuries were observed at 1–4 weeks after IR, including exacerbation of inflammation and extensive alveolitis, the infiltration of multiple lymphocytes, alveolar macrophages and hyaline membrane formation, so that the lung injury score was significantly higher in the BIPF + RILI group compared to the sole IR or BLM treatment (Fig. 2B).

To explore the crucial genes and signaling pathways underlying above severe lung injury, at week-1 after IR, the total RNA of the lung tissues in the severely injured group and its control were collected for sequencing assay. The volcano plot showed 406 upregulated and 382 downregulated genes in the BIPF + RILI group compared to the control (Fig. 2C), and Fig. 2D showed the top fifty DEGs clustered between two groups. GSEA were performed to further analyze the differences of relevant genes expression. Similar to the BIPF or RILI model, the chemokine-mediated signaling pathway and mononuclear migration were positively enriched in the BIPF + RILI model (Fig. 2E). In addition, it was also verified by GO analysis that the transcriptional signals of serious lung injury were enriched in cell migration and chemotaxis of leukocyte and mononuclear cells (Fig. 2F). Collectively, the tissue staining and RNA-seq analysis provided strong evidence that a variety of inflammatory cells were infiltrated to lung tissues during the pathological progression of severe lung injury.

### P21 overexpressed in epithelial MLE-12 and Beae-2B cells after different noxious stimulus

**Fig. 3:**
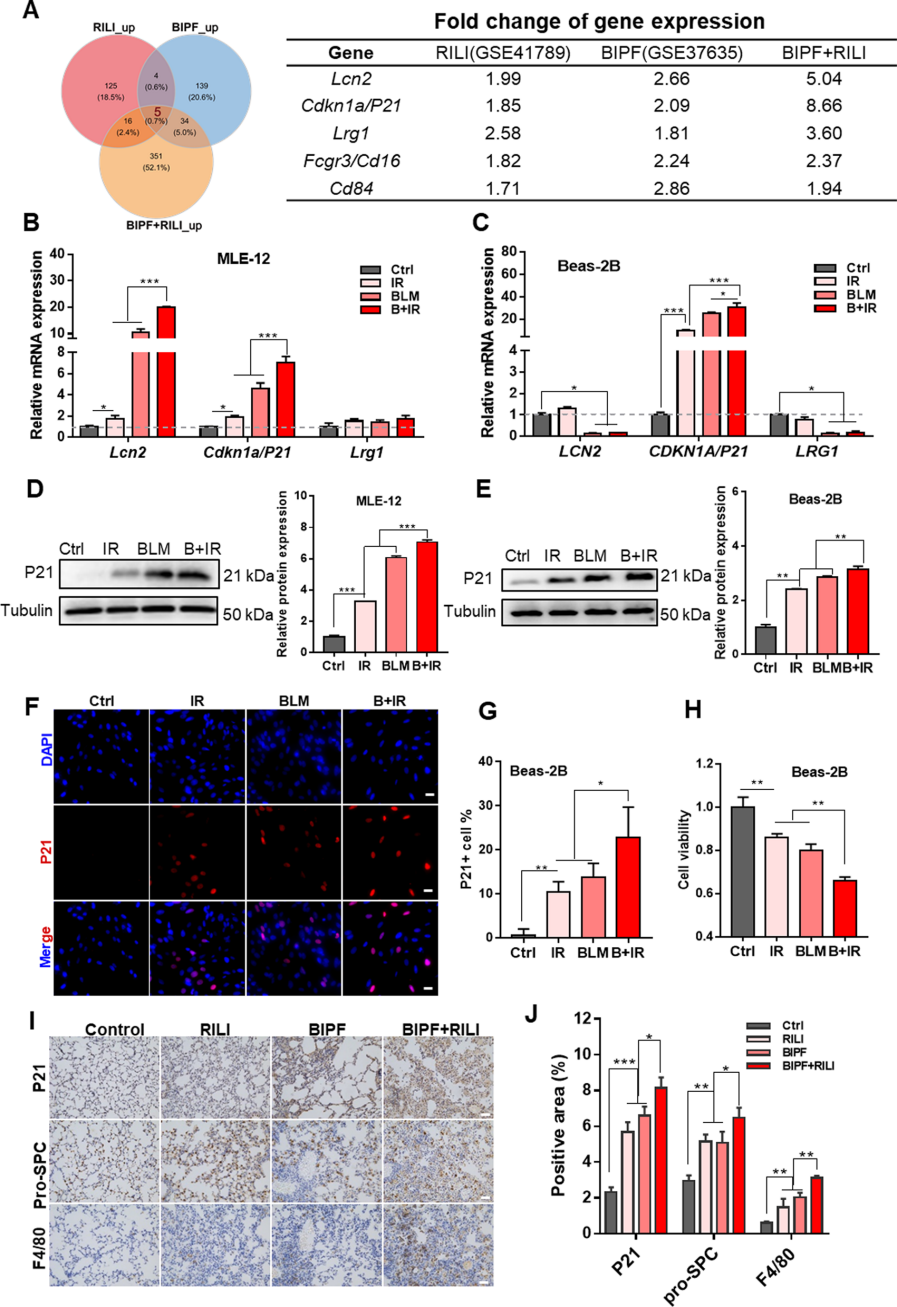
P21 was overexpressed in the RILI, BIPF, and BIPF + RILI model mice. **A** Venn diagram illustrating the intersection of the upregulation genes in three groups of model mice: RILI, BIPF, BIPF + RILI (left), and the gene list and fold change of top 5 common upregulated genes (right). **B**–**C** RNA expression levels of top three genes from Venn diagram **A** in MLE-12 **B** and Beas-2B cells **C** after IR and/or BLM treatment. **D**–**E** Protein expression level of P21 in MLE-12 **D** and Beas-2B cells **E**. **F** Immunofluorescence images of P21 (red color) and DAPI (blue color) in Beas-2B cells. Scale bar = 20 μm.** G** The proportion of P21-positive Beas-2B cells after IR and/or BLM treatment. **H** Viability of Beas-2B cells after IR and/or BLM treatment. **I** Immunohistochemistry images of P21, pro-SPC, and F4/80 in the lung tissues of mice model RILI, BIPF, and BIPF + RILI at week 4 after IR. Scale bar = 50 μm. **J** Positive area of P21, pro-SPC, and F4/80 in the lung tissues of above model mice. *p < 0.05, **p < 0.01, ***p < 0.001

Based on the analyses of microarray datasets and the RNA-seq data of animal models, five intersected DEGs (*Lcn2, Cdkn1a/p21*, *Lrg1, Fcgr3/Cd16* and *Cd84*) were screened out among 150 upregulated DEGs in the RILI model, 182 upregulated genes in the BIPF model, and 406 upregulated genes in the BIPF + RILI model (Fig. 3A). We examined the transcription level of above top three DEGs in both mouse lung epithelial cell line MLE-12 and human lung epithelial cell line Beas-2B, and found that the expression levels of *Lcn2* and *p21* were significantly increased in the IR, BLM and B + IR groups of MLE-12 cells compared to the non-treatment control (Fig. 3B). However, only *p21* gene was significantly upregulated in Beas-2B cells after different treatments (Fig. 3C). Consistently, western blot assay revealed that the expression level of P21 protein was enhanced in both MLE-12 and Beas-2B cells after the treatment of IR, BLM, or BLM plus IR (Fig. 3D, E).

The expression of P21 in Beas-2B cells was also detected by immunofluorescence staining. Its expression in the group B + IR was significantly higher than that in IR- or BLM-treated cells (Fig. 3F). The proportion of P21 positive cells increased from 1% of control to 10.3%, 13.7% and 22.9% in the group of IR, BLM, and B + IR, respectively (Fig. 3G). In addition, CCK-8 assay showed that the viability of Beas-2B cells orderly reduced in the group of IR, BLM, and B + IR (Fig. 3H) and thus was negatively correlated with the expression level of P21.

The bioinformatic analysis indicated that leukocyte chemotaxis might contribute to the progression of lung injury. The role of macrophages, one critical subtype of leukocytes, has got much attention in lung injury [25]. Thus, we intended to investigate the potential link between *p21* in the alveolar epithelial cells and macrophage chemotaxis by immunohistochemically staining P21, AECII marker pro-SPC and macrophage maker F4/80 in the lung tissues of BLM-pretreated mice at week-4 after IR (Fig. 3I). It was found that the P21-positive area mainly located in the fibral niche and thickened alveolar septal of lung, and its percentage was obviously raised to about 6% in the RILI and BIPF mouse models, even up to 8.0% in the severely damaged mouse model (Fig. 3J). The percentage of AECII (pro-SPC-positive) in response to alveolar remodeling and the percentage of macrophages (F4/80-positive) were also increased after BLM and/or IR treatment. Collectively, the overexpression of P21 in epithelial Beas-2B cells was in line with U937 macrophage infiltration in the lung injured by BLM and IR, especially BLM + IR, suggesting that the macrophage recruitment might associate with the overexpression of P21 in lung epithelial cells.

### Stressed epithelial Beas-2B cells facilitated M1 polarization of macrophages

**Fig. 4:**
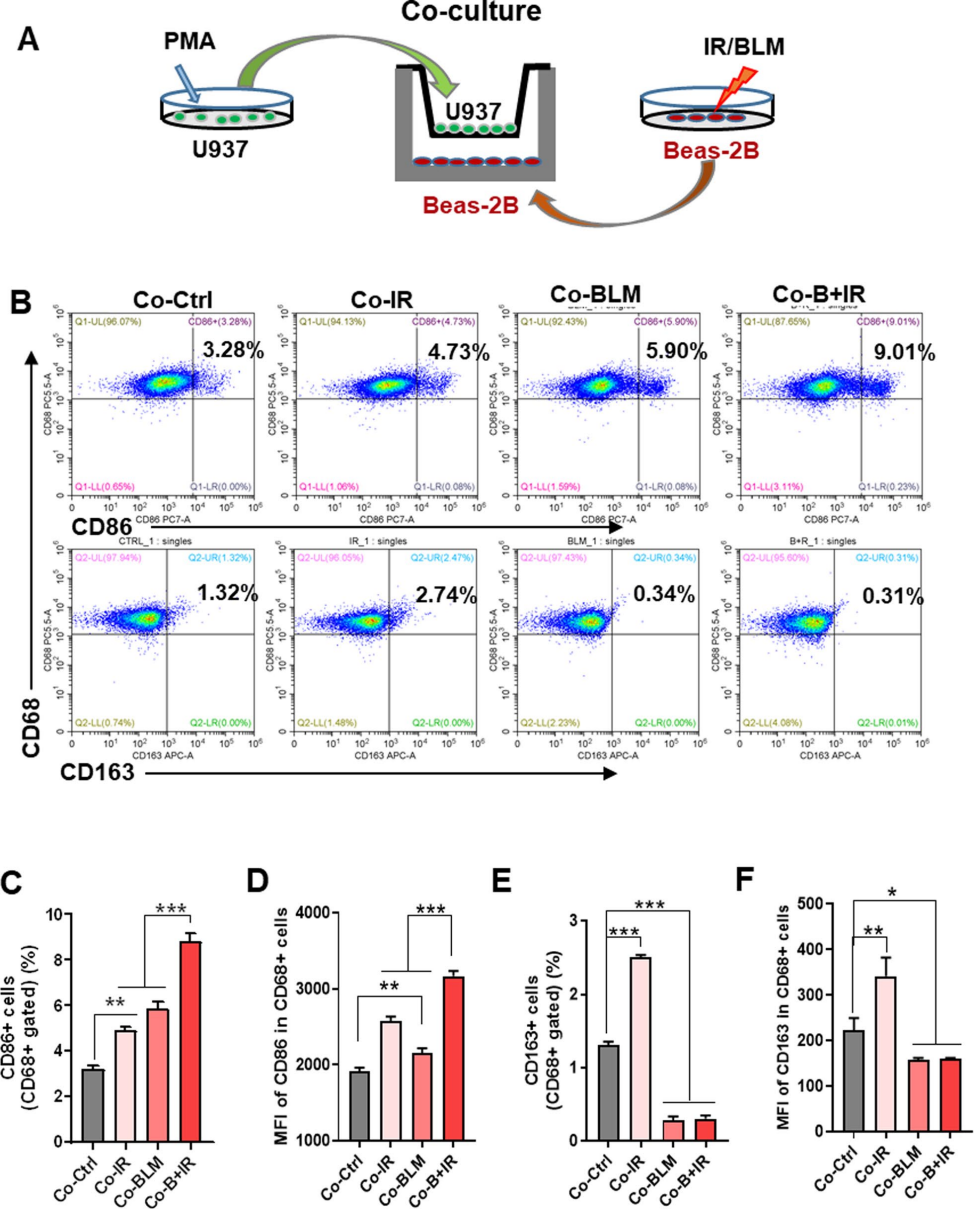
The stress-injured epithelial Beas-2B cells facilitated M1-polarization of macrophages. **A** A co-culture system of Beas-2B cells treated with IR and/or BLM and PMA-pretreated U937 cells. **B** Flow cytometric analysis of the ratio of CD86 + (M1 marker) and CD163 + (M2 marker) macrophages within above co-cultured U937 cells. **C**-**D** The percentage **C** and mean fluorescence intensity (MFI) **D** of CD86 + cells in CD68 + population within the above co-cultured U937 cells. **E**–**F** The percentage **E** and MFI value **F** of CD163 + cells in CD68 + population within the above co-cultured U937 cells. *p < 0.05, **p < 0.01, ***p < 0.001

To explore the intercellular communication between the stressed epithelial Beas-2B cells and U937 macrophages, a cell co-culture system was used in this study (Fig. 4A). PMA-pretreated U937 were planted in the upper insert dish, and Beas-2B cells pre-treated with IR and/or BLM were seeded in the lower chamber. Considering the phenotype and function of macrophages are highly plastic to different stimulation, the macrophages in the co-culture system were collected to detect their polarization using flow cytometry. It was found that the quantity of M1 macrophages (CD86-positive) was significantly increased after co-culture with stressed Beas-2B cells (Fig. 4B, C), e.g., it had a maximum value of 9.0% in the group B + IR. In addition, the mean fluorescence intensity (MFI) of CD86 protein in CD68-positive cells (M0 macrophages) was increased more obviously in group B + IR than that in the single treatment groups (Fig. 4D). Meanwhile, the proportion of positively expressed M2 macrophages marker CD163 and the MFI of CD163 protein in CD68-positive cells were also raised after co-culture with IR-treated Beas-2B cells compared with control, which was decreased after co-culture with BLM-treated epithelial cells or B + IR treatments (Fig. 4E, F).

### Overexpressing P21 in epithelial Beas-2B cells promoted chemotaxis of macrophages

**Fig. 5:**
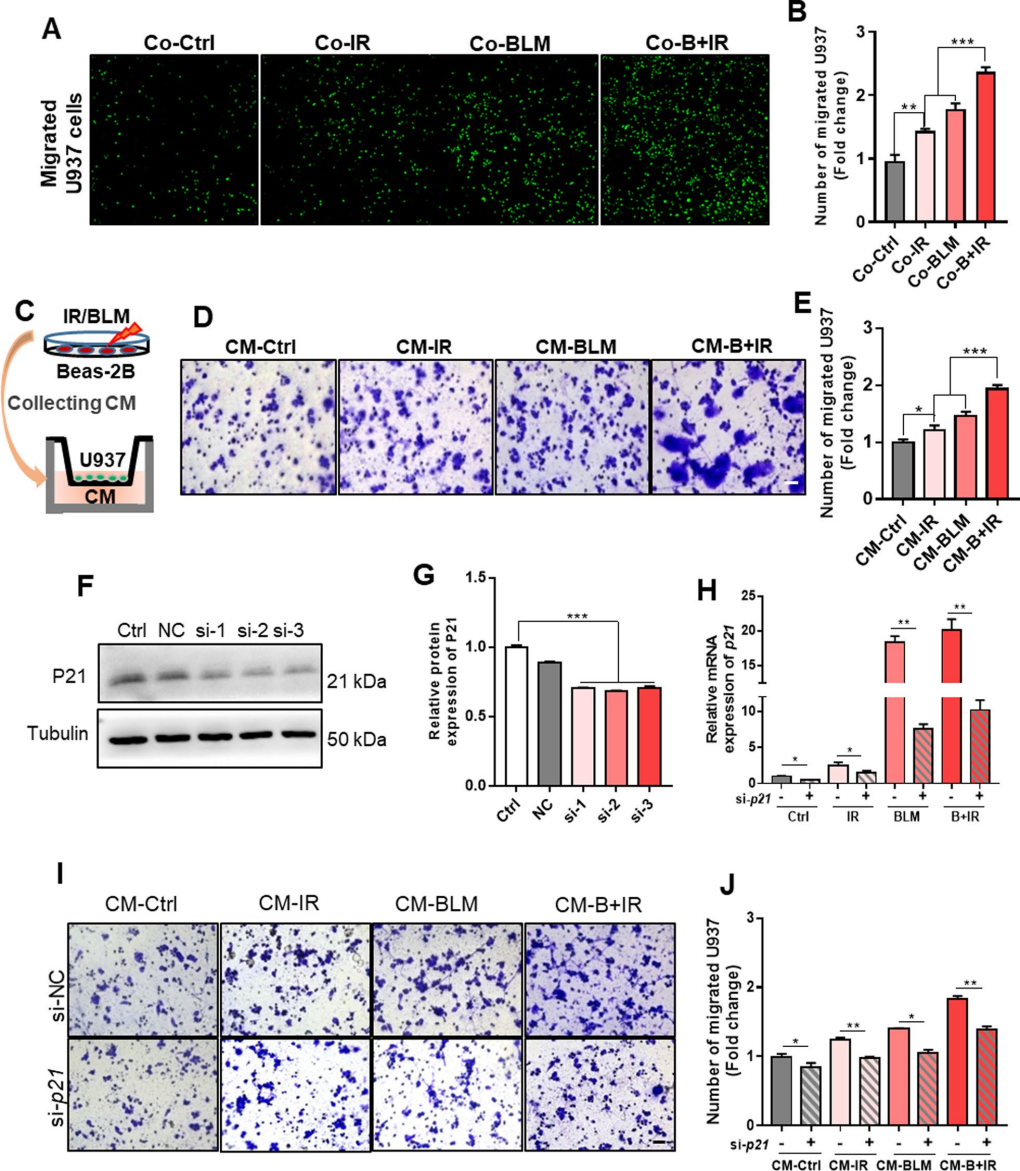
Knockdown *p21* in Beas-2B cells reduced the chemotaxis of U937 macrophages. **A**–**B** Representative images **A** and number **B** of migrated U937 cells after 24 h co-culture with Beas-2B cells treated with IR, BLM, or BLM + IR. Scale bar = 50 μm. **C** A scheme of the conditional medium (CM) generation. At 24 h after the treatment of IR and/or BLM, the supernatant of Beas-2B cells was collected as CM. **D**-**E** Representative images **D** and the number **E** of migrated U937 cells after 48 h treatment of the CM with respect to IR and/or BLM treatment. Scale bar = 50 μm. **F**-**G** Western blot assay of P21 in Beas-2B cells after *p21-*siRNA transfection. **H** The expression level of *p21* mRNA in Beas-2B cells treated with IR and/or BLM. **I**–**J** Representative images **I** and the number **J** of migrated U937 cells after 48 h treatment of CM from *p21*-siRNA transfected Beas-2B cells treated with IR and/or BLM. Scale bar = 50 μm. *p < 0.05, **p < 0.01, ***p < 0.001

To identify whether the stressed epithelial Beas-2B cells could promote the chemotaxis of U937 macrophages, cell migration assay was conducted. It was found that the migration ability of U937 cells was gradually increased by the Beas-2B cells pretreated with IR, BLM and B + IR after co-culture for 24 h (Fig. 5A, B). Combining with the result in Fig. 3H showing that the survival of Beas-2B cells decreased along with the treatment of IR, BLM and B + IR, it could be deduced that the ability of chemotaxis macrophages increased along with the damage degree of bystander epithelial cells. To confirm whether it was the secretion factors of the stressed epithelial cells recruiting the macrophages, the supernatant of Beas-2B cells after IR and/or BLM treatment was collected as the conditional medium (CM) to treat U937 cells in a co-culture system (Fig. 5C). After culturing with this CM for 48 h, the number of migrated U937 cells was also significantly increased to 1.2-, 1.5- and 1.9-fold of control with respect to the IR-, BLM-, and B + IR-treated Beas-2B cells, respectively (Fig. 5D, E).

As *p21* being significantly upregulated in both stressed epithelial cells in vitro and mouse lung in vivo, we further investigated the relationship between *p21* and macrophage chemotaxis. When Beas-2B cells were transfected with *p21-*siRNA with a knockdown efficiency of about 50% (Fig. 5F, G) before receiving different treatments, the expression levels of *p21* mRNA in Beas-2B cells treated by IR, BLM, or B + IR were all reduced (Fig. 5H). Meanwhile, the CM-induced migration of U937 cells was also reduced to 70%-80% of control in each group (Fig. 5I, J). These results indicated that *p21* participated in promoting macrophage chemotaxis by secreting some signaling factors into CM.

### P21 mediated U937 macrophage chemotaxis by CCL7

**Fig. 6:**
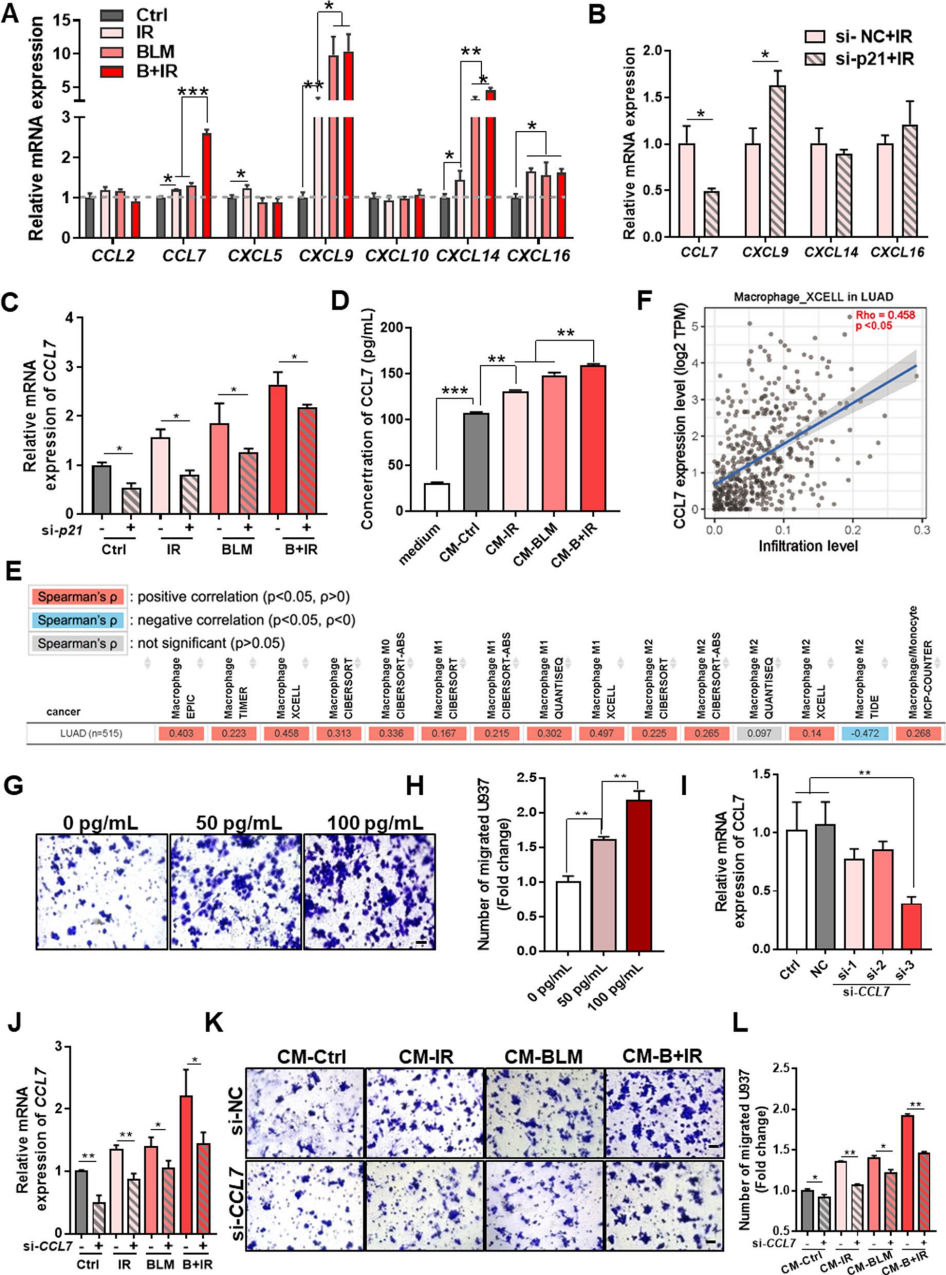
P21-mediated CCL7 release from injured Beas-2B cells promoted U937 macrophage chemotaxis. **A** RNA expression levels of some chemokines in Beas-2B cells after IR and/or BLM treatment.** B** RNA expression levels of chemokines in the irradiated Beas-2B cells with/without *p21*-siRNA interference. **C** RNA expression level of *CCL7* in *p21*-siRNA transfected Beas-2B cells after IR and/or BLM treatment. **D** CCL7 concentration in the supernatants of Beas-2B at 24 h after the treatment of IR and/or BLM. **E**–**F** The correlation between *CCL7* gene expression level and macrophage infiltration in lung adenocarcinoma (LUAD) in databases integrated by Timer2.0 **E** and the specifics in the XCELL database **F**. **G**-**H** Representative images **G** and the number **H** of migrated U937 cells cultured in a medium containing different concentrations of active CCL7 protein. Scale bar = 50 μm. **I** qRT-PCR assay of *CCL7* in Beas-2B cells after *CCL7-*siRNA transfection. **J** The expression level of *CCL7* mRNA in Beas-2B cells treated with IR and/or BLM after *CCL7*-siRNA transfection. **K-L** Representative images **K** and the number **L** of migrated U937 cells after 48 h treatment of CM from *CCL7*-siRNA transfected Beas-2B cells treated with IR and/or BLM. Scale bar = 50 μm. *p < 0.05, **p < 0.01, ***p < 0.001

Chemokines, consisting of a large number of ligands and receptors, participate in the function and organization of the immune system [26]. To identify the critical signaling factors that may contribute to the chemotaxis of macrophages in the lung injury after different stresses, the expressions of some chemokines associated with monocytes, including *CCL2*, *CCL7*, *CXCL5*, *CXCL9*, *CXCL10*, *CXCL14*, and *CXCL16* were examined in Beas-2B cells. It was found that the mRNA expression levels of *CCL7*, *CXCL9*, *CXCL14*, and *CXCL16* were elevated in the cells after IR and/or BLM treatment (Fig. 6A). When *p21* was knocked-down in Beas-2B cells, only the expression level of *CCL7* was significantly reduced in the cells treated by IR and/or BLM (Fig. 6B, C), which indicated that *p21* and *CCL7* had a positive relationship in the stress responses. In addition, at 24 h after IR, the concentration of CCL7 protein in the supernatant of Beas-2B cells increased from 100 pg/mL of control to 130 pg/mL, 145 pg/mL and 159 pg/mL in the group of IR, BLM, and B + IR, respectively (Fig. 6D).

Moreover, the relationship between *CCL7* and macrophage infiltration was investigated. Analysis of seven databases of lung adenocarcinoma (LUAD) integrated by Timer 2.0 illustrated that the *CCL7* expression level positively correlated with the infiltration of macrophages including M0, M1 and M2 phases in six databases (Fig. 6E). As an example, in the XCELL database, there was a positive correlation coefficient between *CCL7* and macrophage infiltration (Fig. 6F), suggesting the recruitment function of CCL7 in macrophage chemotaxis. On the other hand, in vitro cell migration assay further illustrated that the treatment of U937 cells with exogenous CCL7 protein could promote the migration of macrophages in a concentration-dependent manner (Fig. 6G, H). Oppositely, when Beas-2B cells were transfected with *CCL7*-siRNA with a knockdown efficiency of about 60% (si-*CCL7*-3) and then treated with IR, BLM, or B + IR (Fig. 6I, J), the number of chemotactic U937 cells was significantly reduced after co-culturing with the CM of above Beas-2B cells, especially in the group B + IR (Fig. 6K, L). Taken together, IR and BLM up-regulated *p21* in the lung epithelial Beas-2B cells, stimulated the production of CCL7, and thus promoted the chemotaxis of U937 macrophages.

## Discussion

In our study, a comparative analysis of microarray data from two mouse lung injury models was performed at transcriptome level between RILI and BIPF. Moreover, a one-shot IR induced severe lung injury mouse model based on pre-existing ILDs was established to further identify the mechanisms through RNA-seq data analysis. Bioinformatic analysis revealed the commonalities and similarities underlying the development of lung injury after exposure to different stimuli. Inflammatory responses, including cell chemotaxis and cytokine secretion, and fibrosis-related signaling pathways including extracellular matrix (ECM), CAMs and PI3K-AKT, were all enriched in DEGs of three models. Especially, the chemokine signaling pathway, cytokine-cytokine receptor interaction and immune system process were essential in the lung toxicity after IR, BLM or BLM plus IR treatment (Figs. 1 and 2). Actually, immune system could be initially activated to maintain microenvironment homeostasis of macrophages, lymphocytes and granulocytes, which was characterized by multiple immune cells infiltration and a series of cytokines and chemokines [26]. However, imbalance between inflammation and restoration responses contributes to the worsening of lung injury from pneumonitis to pulmonary fibrosis, and their potential biomarkers including inflammation-related factors (IL-1β, IL-6, IL-8, IL-10 and CRP) and fibrosis-related factors (TGF-β, ET-1, KL-6, and PAI-1) have been reviewed previously [27].

Pulmonary is one of primary targets of numerous drugs. ILDs are the most common type of drug-induced lung injury (DILI) and can be caused by more than 300 drugs, which account for about 70% of DILIs [1]. It was reported that 5–10% of lung cancer patients suffered with ILDs [28]. When the patients with serious lung diseases received thoracic radiotherapy, the risk of treatment-relevant lung toxicities including fatal radiation pneumonitis (0–30%) could be increased despite the application of precise radiotherapy techniques in clinical [29]. The high incidence of acute exacerbation has been observed in the patients with preexisting ILDs after radiotherapy or chemotherapy. Especially, when chemoradiotherapy was delivered, the rate of severe radiation pneumonitis raised up to 46% [30, 31]. Using our established animal model, it was found that IR could induce severe diffuse alveolar damage (DAD) in the lung preexisting interstitial abnormalities. Acute exacerbation of inflammation was monitored with the infiltration of multiple lymphocytes, thickened alveolar septum, myofibroblast hyperplasia, fibrinous exudate and collagen deposition (Fig. 2). High grade (≥ 3) lung toxicities especially pulmonary fibrosis are often irreversible, and the severe cases can result in respiratory failure or even death.

By using single-cell transcriptome analysis, the percentages of immune cells in lung tissues were determined at the acute phase after IR. Significantly increased macrophages and decreased B, T, and NK cells were observed in comparison with healthy lungs [32]. It is known that macrophages function as important components of innate immune system, tissue homeostasis and pathogen elimination. Several distinct macrophage populations in the lung were identified as tissue-resident alveolar macrophages (TR-AMs), lung tissue-resident interstitial macrophages (TR-IMs) and monocyte-derived alveolar macrophages (Mo-AMs) [33]. Infiltrating macrophages appeared to play a particularly important role in lung injury. Specific inhibition of Mo-AMs’ function or deletion of tissue infiltrating macrophages could ameliorate BLM-induced lung fibrosis, whereas TR-AMs could not [6, 34]. Moreover, Mo-AMs could provide a link between epithelial injury and activation of resident fibroblasts [35]. Here, we found that IR and/or BLM-stressed lung epithelial Beas-2B cells could promote the chemotaxis of and macrophage U937 cells, and the recruitment rate was positively correlated with the damage degree of Beas-2B cells. Additionally, the injured epithelial cells favored to promote macrophages polarization to the activated phase M1. In acute phase of lung damage, M1 macrophages performed pro-inflammatory responses via producing inducible nitric oxide synthase (iNOS), TNF-α, IL-1β, IL-6, IL-15, IL-12 and IL-23 [36, 37].

Additionally, the driven role of dysfunctional AECs in respiratory impairment has become a consensus. AECII acts as stem cells in maintaining alveolar regeneration by trans-differentiating into AECI [38, 39]. During the period of lung fibrosis, several intermediate AECII was detected by specific markers such as keratin 8 (KRT8), claudin 4 (CLDN4) and stratifin (SFN). When the alveolar repair failed due to repetitive injury, the progression of chronic lung diseases could be triggered [40]. It was demonstrated that radiation-induced AECII senescence participated in pulmonary fibrosis, and NADPH oxidase (NOX) and insulin-like growth factor-1 receptor (IGF-1R) were significantly involved in [41, 42]. Furthermore, the interaction between epithelial cells and stromal cells in the microenvironment are complex through a variety of ways including signaling mediators, extracellular vesicles, membrane glycoproteins and their receptors, and gap junction channels [7, 43]. To further investigate how the stressed epithelial cells interacted with macrophages, we examined the expression levels of the common DEGs in three lung damage mouse models. The cell cycle inhibition regulating gene *Cdkn1a/p21* was screened out in vivo, and was further verified to be elevated at both transcription and protein levels in lung epithelial cell lines (MLE-12 and Beas-2B) after IR and/or BLM treatment (Fig. 3), whose upregulation was consistent with the degree of lung damage.

P21 is a direct target of phosphorylated-P53. Its functions as G1-phase arrest and a critical marker of cellular senescence have been well recognized [44]. Recently, *p21* upregulation was found in a time-dependent manner in the BIPF of mice, and AECII undergoing *p21*-mediated senescence lost their abilities in self-renew and differentiate in association with the development of fibrotic lung diseases [10]. On the contrary, as an influenza restriction factor, the protective effect of *p21* was observed in a *p53*-independent way, where the antiviral activity of *p21* was accomplished though promoting interferon regulatory factor 3 (IRF3) activation and increased the expression of type I interferons (IFNs) [45]. Moreover, it was demonstrated that *p21* could place the stressed-cells under immunosurveillance by attracting macrophages and recruiting cytotoxic T lymphocytes. P21-activated secretory phenotype (PASP) showed a prominent role in protecting the stressed-cells from neoplastic transformation through CXCL14-dependant macrophage attraction [46]. In our study, CXCL14 was also upregulated in the injured epithelial cells along with other monocytes chemotaxis-related chemokines CCL7, CXCL9, and CXCL16. However, our study demonstrated that *p21*-dependant CCL7 secretion in the epithelial cells played more an essential role in macrophage chemotaxis. When the expression of *p21* was inhibited, the infiltration of macrophages was reduced along with *CCL7* downregulation, suggesting the immunologic surveillance role of *p21* in the lung injury through CCL7 secretion after various stimuli of IR and/or BLM (Figs. 5 and 6).

**Fig. 7:**
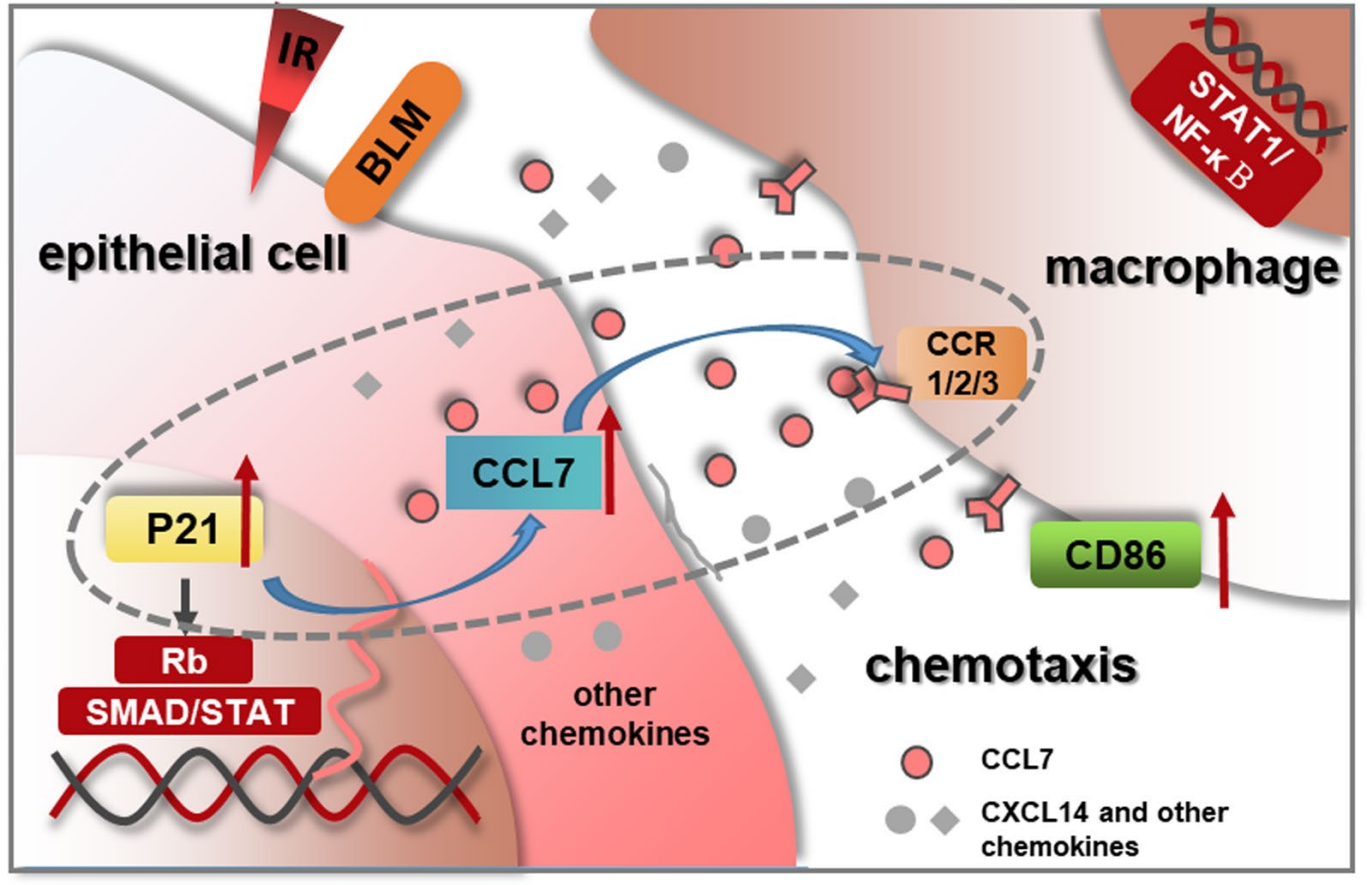
The schematic model of P21-mediated chemotaxis of macrophages by CCL7 secretion from injured epithelial cells. After IR or BLM exposure, P21 was upregulated in stressed epithelial cells and then promoted CCL7 production and secretion. Macrophages were in turn recruited by CCL7 chemokine via binding to their receptors. Polarization of M1 macrophages functioned as inflammatory effects through NF-κB/STAT1 pathway

CCL7 was reported as one of inflammatory chemokines in the anti-infectious immunity, where monocytes, macrophages and neutrophils were recruited for viral clearance in the infected tissues and organs [26]. In addition, it was found that the overexpression of CCL7 in tumor cells could facilitate the anti-PD-1 therapy in lung cancer via recruiting DC1 cells into the tumor immune microenvironment [47]. Besides immune cell recruitment, CCL7-mediated cellular proliferation and activation were also demonstrated. Accumulating evidence suggested that the progression and exacerbation of lung fibrosis was relevant to CC chemokines and CXC chemokines, whereas its potential roles in diagnosis and targeted treatment need further evaluation [48]. In this study, IR/BLM-induced CCL7 chemokine in the epithelial cells was *p21*-dependent and contributed to macrophage recruitment in vitro (Fig. 7). Similarly, studies have revealed that overexpressed P21 in epithelial cells mediated immunosurveillance after exposure to different stimuli, and the interaction between P21-activated retinoblastoma protein (Rb) with signal transducer and activator of transcription (STAT) and SMAD transcription factors at selected gene promoters were involved in promoting the production of various cytokines or chemokines [46, 49]. The expression level of chemokine CCL7 was increased by P21 in our experiments, and CCL7 secreted by stressed epithelial cells in turn recruited macrophages via binding to CCR1/2/3 or CCR5/10 [50, 51]. Meanwhile, infiltrated macrophages were polarized towards M1 phenotype, which would function as pro-inflammatory effects through the activation of NF-κB/STAT1 signaling pathways [52, 53].

Based on the innovatively established mouse models and RNA-seq data analysis, this study explored the common signaling pathway in the lung injury induced by different toxic factors and revealed the role of P21-dependent CCL7 protein in macrophage chemotaxis in vitro. Restricting the infiltration of macrophages into the lung tissues can attenuate pulmonary fibrosis by limiting collagen production and fibrotic remodeling in the BIPF model [34, 54]. Drugs targeting CCL7 may not only be used in the treatment of lung cancer but also alleviate lung injury caused by different toxins [47]. More in vivo experiments with knockout model and neutralization antibodies are needed to further confirm the role of CCL7 in lung injury for translating this finding into clinical practice. In the future, it is critical to determine which type of cells can secrete CCL7 chemokine promoting the infiltration of macrophages in the lung.

## Conclusions

This study elucidated the common pathophysiology of immune cell infiltration during severe lung injury after IR and/or BLM treatment, and disclosed the signaling factors underlying the intercellular communication between the damaged epithelial cells and immune macrophages via P21-CCL7 axis in vitro. Actually, the persistent activation of immune system and the imbalance between damage and repair processes accounted for the irreversible changes of alveolar structure, leading to subsequent pneumonitis and pulmonary fibrosis. The complex intercellular communications in the immune microenvironment during lung injury remain to be addressed progressively. Further studies are needed to exploit the common or specific molecular mechanisms associated with immune microenvironment in injured lung after exposure to physical and/or chemical toxins such as irradiation and bleomycin.

## Data Availability

Data presented in this study are available on request from the corresponding author upon reasonable request. The public datasets GSE41789 and GSE37635 supporting the conclusions of this article are available in GEO database (https://www.ncbi.nlm.nih.gov/geo/).

## Abbreviations

ILDs: Interstitial lung diseases
BLM: Bleomycin
BIPF: Bleomycin-induced pulmonary fibrosis
RILI: Radiation-induced lung injury
AECs: Alveolar epithelial cells
IR: Irradiation
GEO: Gene expression omnibus
DEGs: Differentially expressed genes
GO: Gene ontology
KEGG: Kyoto encyclopedia of genes and genomes
DAVID: The database for annotation, visualization, and integrated discovery
H&E: Hematoxylin–eosin staining
GSEA: Gene set enrichment analysis
FBS: Fetal bovine serum
PMA: Phorbol 12-myristate 13-acetate
PBS: Phosphate buffered saline
BP: Biological process
MF: Molecular function
CC: Cellular component
MFI: Mean fluorescence intensity
CAMs: Cell adhesion molecules
CM: Conditional medium
LUAD: Lung adenocarcinoma
ECM: Extracellular matrix
DILI: Drug-induced lung injury
DAD: Diffuse alveolar damage
TR-AMs: Tissue-resident alveolar macrophages
TR-IMs: Tissue-resident interstitial macrophages
Mo-AMs: Monocyte-derived alveolar macrophages
PASP: P21-activated secretory phenotype
